# Efficacy of Venner-PneuX endotracheal tube system for prevention of ventilator-associated pneumonia in intensive care units: A protocol for systematic review and meta-analysis

**DOI:** 10.1097/MD.0000000000024278

**Published:** 2021-01-08

**Authors:** Min Gan, Zhuming Bao, Juan Han

**Affiliations:** a Medical Faculty, Yunnan University of Business Management; b Faculty of Nursing, Yunnan Medical Health College, 296 Haitun Road, Kunming Yunnan, P.R. China; c Faculty of Nursing, Roseleigh, University College Cork, Cork, Ireland.

**Keywords:** intensive care units, protocol, systematic review, venner-PneuX endotracheal tube system, ventilator-associated pneumonia

## Abstract

**Background:**

The pathogenic mechanism and prevention of ventilator-associated pneumonia (VAP) are substantially improved over the past several decades, but VAP remains frequently seen among the critical cases. The Venner-PneuX endotracheal tube system (VPXETS) has been proved to perform better than standard endotracheal tubes (SET) in the prevention of VAP in some studies. Therefore, this systematic review is aimed at evaluating the effectiveness of VPXETS in order to prevent VAP.

**Methods:**

Electronic databases, including PubMed, WANFANG, CENTRAL, CNKI, EMBASE, and CINAHL, are used to search relevant randomized controlled trials for evaluating the therapeutic effect of VPXETS on preventing VAP from January 2011 to December 2020. To be specific, related studies are selected, data are extracted, risk of bias is assessed, and meta-analysis is conducted in succession.

**Results:**

The present review aims to assess the therapeutic effect of VPXETS on preventing VAP in intensive care units (ICUs). Our outcome measures include the incidence and side reaction of VAP.

**Conclusions:**

The present review assesses related studies regarding the therapeutic effect of VPXETS on preventing VAP at ICUs.

**Dissemination and ethics:**

Our findings in this work are to be disseminated by means of peer-reviewed publication. No ethical approval is required in our review since it uses the published data. Moreover, anonymity is guaranteed during the data analysis process.

**OSF Registration number:**

DOI 10.17605/OSF.IO/6BERJ

## 1 Introduction

Ventilator-associated pneumonia (VAP) refers to the pneumonia among cases requiring a certain device for assisting or controlling the continuous respiration via the tracheostomy tube or endotracheal tubes (ETs) in 48 before infection onset.^[1]^ VAP, one of the commonly seen complications secondary to mechanical ventilation (MV), is possibly a frequently seen infection that takes place at intensive care units (ICUs).^[2,3]^ At ICUs, the VAP risk is 9% to 27%, and the incidence is 5 to 10 patients/1000 ventilator days.^[4]^ VAP has a high morbidity and leads to increased medical expenditures and extended hospital stay. According to the estimation, every VAP episode costs up to $40,000.^[5]^ Additionally, it will also elevate the mortality rate to up to 20% to 60%.^[5]^ Based on these figures in mind, the prevention of VAP provides paramount importance.

At present, VAP is most commonly caused by the inhalation of the infectant excreta produced by the oropharyngeal space.^[6]^ For critical patients, the risk factors for inhalation of oropharyngeal excreta are accumulation on ET cuff, reintubation, upper airway colonization by nosocomial pathogenic bacteria, unsuitable ET cuff pressure, biofilm formation, and bacterial colonization within the ET lumen.^[7]^ The Venner-PneuX endotracheal tube system (VPXETS) has incorporated multiple strategies for minimizing the inhalation of oropharyngeal excreta, which is approved in the UK to be used among cases requiring intubation or MV in the treatment.^[8]^ Such ETs contain the subglottic suction ports for sucking excreta accumulated within the subglottic space. In these ETs, the low-pressure and low-volume cuff can inflate in the absence of creases or folds, and the tracheal seal monitor can keep the best cuff pressure. Besides, these ETs have nonstick lining, which can prevent the adherence of microorganisms and the formation of bio-films. Moreover, certain studies suggest that VPXETS outperforms the standard endotracheal tubes (SET) in the prevention of VAP.

As a result, the current review aims to systematically review all randomized controlled trials (RCTs) to evaluate the effectiveness of VPXETS for preventing VAP in critically ill patients.

## 2 Materials and methods

The present systematic review protocol was registered on OSF on December 10, 2020 (Registration number: DOI 10.17605/OSF.IO/6BERJ), which was designed in accordance with the Cochrane Handbook for Systematic Reviews of Interventions and the Preferred Reporting Items for Systematic Reviews and Meta-Analysis Protocol (PRISMA-P) guidelines.^[9]^ Any change in the present review is to be described when necessary.

## 3 Study inclusion criteria

### 3.1 Study type

Relevant RCTs associated with the use of VPXETS in preventing VAP among the critical cases will be enrolled in the present work, with no restriction on publication status or language. In addition, the quasi-RCTs, non-RCTs, case series, case reports, uncontrolled trials, cross-over articles, or laboratory studies are eliminated from this review.

### 3.2 Subject type

Subjects receiving MV for >48 hours at ICUs were collected despite the age, sex, or race.

### 3.3 Intervention type

Interventions adopted in this work include VPXETS, which is constituted by 1 ET equipped with the irrigation and subglottic suction ports together with one port for attaching the Venner Tracheal seal monitor. RCTs that include VPXETS in conjunction with additional interventions (like diverse oral care types or head up positions) are excluded. The SETs are used for control. All cases should receive identical routine respiratory care at ICUs in line with the ventilator care package in both intervention and control groups.

### 3.4 Outcome measure type

#### 3.4.1 Primary endpoint (s)

The VAP incidence is selected as the primary endpoint.

#### 3.4.2 Secondary endpoint (s)

The lengths of ICU and hospital stays are the secondary endpoints.

## 4 Study retrieval and identification methods

### 4.1 Searches against the electronic databases

Electronic databases shown below are searched to identify related RCTs:

1. CNKI (China National Knowledge Infrastructure Database, from 2011 to present);
2. WANFANG Database (from 2011 to present);
3. PubMed Database (from 2011 to present);
4. CENTRAL (Cochrane Central Register of Controlled Trials, from 2011 to present);
5. CINAHL (Cumulative Index of Nursing and Allied Health Literature, from 2011 to present);
6. EMBASE (Excerpta Medica database, from 2011 to present);
7. Ovid MEDLINE ALL (Ovid Medical Literature Analysis and Retrieval System Online, from 2011 to present).

Additionally, the Clinical trial registries, including the Netherlands National Trial Register (NTR), the Chinese Clinical Trial Registry (ChiCTR), as well as the ClinicalTrials.gov, are searched to identify those ongoing yet unpublished trials.

RCTs are screened without any restriction on language.

### 4.2 Data extraction and analysis

#### 4.2.1 Study selection

The EndNote X9 software is employed for managing records from the electronic databases searched. To be specific, study titles and abstracts will be selected. Later, full text of related articles will be examined by 2 reviewers (MG and ZB) according to our inclusion criteria. Afterwards, these 2 reviewers will review the related studies to decide whether they conform to the set criteria, and the disagreements between them are settled down by the opinion of a third review. Studies are selected following our selection procedure and recorded into the PRISMA flow chart. Thereafter, The Grading of Recommendations Assessment, Development and Evaluation (GRADE) will be used for evaluation.

#### 4.2.2 Data extraction and analysis

In line with our inclusion criteria, we will prepare a standard data extraction form to collect data. Specifically, the data shown below are collected by 2 reviewers (MG and ZB):

1. *General data:* study identify, year of publication, title and first author;
2. *Study methodology:* study design, concealment of allocation randomization, sample size, blinding, insufficient data or selective report, additional bias sources;
3. *Participants:* Study inclusion and exclusion criteria;
4. *Intervention:* Details of types;
5. *Control:* Details of types;
6. *Outcomes:* Outcome measures included.

#### 4.2.3 Risk of bias assessment

Two reviewers (MG and ZB) independently evaluate the risk of bias among the enrolled studies by using Cochrane Risk of Bias Tool, and the disagreement between them will be settled down by mutual negotiation or reaching a consensus by a third reviewer. Each judgment is comprehensively depicted, whereas relevant conclusions are made and displayed in Risk of Bias figures and used in combination to interpret the review results through sensitivity analysis. To be specific, in every domain, the risk of bias is classified into inadequate, adequate, or unclear. In this review, the concealment of allocation is graded to investigate the possible heterogeneity in sensitivity analysis. Besides, more study quality aspects include blinding extent (when necessary), noncompliance, loss to follow-up, standardization of outcome assessment, and intention-to-treat (ITT) analysis application, which are displayed in Risk of Bias Table that describes all enrolled articles and they can offer a context to discuss our result creditability.

#### 4.2.4 Data analysis

Stata Software (version 15.1) is used for meta-analyses. On the contrary, the weighted mean difference is utilized to compare continuous variables, and then we will integrate the pooled statistical effects of the two. *χ*^2^ test is used for analyzing the potential heterogeneity regarding every research question enrolled, where *I*^2^ > 50% indicates significant judgment and a random effect model is used; otherwise *I*^2^≤ 50% indicates homogeneity among the enrolled studies and a fixed effect model is adopted. On the contrary, effect size will be presented in the manner of 95% confidence interval, where a difference of *P* < .05 indicates statistical significance. Sensitivity analysis is conducted to examine whether the heterogeneity exists when there is at least 1 outlier study that has conflicting results with others and to exclude the outlier studies. Furthermore, sensitivity analysis will also be conducted for exploring the trial quality effect on the effect estimates. In terms of methodology, its quality components are concealment of allocation, sufficient production of allocation sequences, and the application of (ITT) analysis.

Meta-regression analysis is conducted when they are sufficient data collected.

#### 4.2.5 Bias of publication

Funnel plots (effect size as a function of standard error) will be generated when there are enough trials retrieved (>10), so as to explore the bias of publication.

#### 4.2.6 Ethical statement and dissemination

All data utilized in the present review are extracted from published articles; therefore, ethical approval is waived.

## Author contributions

**Conceptualization:** Min Gan, Zhuming Bao, Juan Han.

**Data curation:** Min Gan, Zhuming Bao.

**Formal analysis:** Min Gan, Zhuming Bao.

Funding acquisition: Min Gan; Juan Han

**Investigation:** Min Gan, Zhuming Bao.

**Methodology:** Min Gan, Juan Han.

**Project administration:** Juan Han.

**Resources:** Min Gan, Juan Han.

**Software:** Juan Han.

**Supervision:** Juan Han.

**Validation:** Juan Han.

**Visualization:** Juan Han.

**Writing – original draft:** Min Gan.

**Writing – review & editing:** Min Gan, Zhuming Bao, Juan Han.

## Corrections

When originally published, the affiliations were labeled in the incorrect order (a, c, b) and have since been corrected (a, b, c). In affiliation a, Yunnan University of Business Management was incorrectly written as Yunnan College of Business Management and has since been corrected.
